# The training pathway for residents: ‘Robotic Curriculum for young Surgeons’ (RoCS) does not impair patient outcome during implementation into clinical routine

**DOI:** 10.1007/s11701-024-02056-9

**Published:** 2024-08-06

**Authors:** Jessica Stockheim, S. Andriof, M. Andric, S. Al-Madhi, S. Acciuffi, M. Franz, E. Lorenz, S. Peglow, F. Benedix, A. Perrakis, R. S. Croner

**Affiliations:** 1 Department of General, Visceral, Vascular, and Transplant Surgery, Otto-von-Guericke University, Magdeburg, Germany; 2 Medical Faculty, University Magdeburg, Magdeburg, Germany

**Keywords:** Oncology, General surgery, Training, Robotics, Robotic surgery

## Abstract

The “Robotic Curriculum for young Surgeons” (RoCS) was launched 03/2020 to address the increasing importance of robotics in surgical training. It aims to provide residents with foundational robotic skills by involving them early in their training. This study evaluated the impact of RoCS’ integration into clinical routine on patient outcomes. Two cohorts were compared regarding the implementation of RoCS: Cohort 1 (before RoCS) included all robot-assisted procedures between 2017 and 03/2020 (*n* = 174 adults) retrospectively; Cohort 2 (after RoCS) included all adults (*n* = 177) who underwent robotic procedures between 03/2020 and 2021 prospectively. Statistical analysis covered demographics, perioperative parameters, and follow-up data, including mortality and morbidity. Subgroup analysis for both cohorts was organ-related (upper gastrointestinal tract (UGI), colorectal (CR), hepatopancreaticobiliary system (HPB)). Sixteen procedures were excluded due to heterogeneity. In-hospital, 30-, 90-day morbidity and mortality showed no significant differences between both cohorts, including organ-related subgroups. For UGI, no significant intraoperative parameter changes were observed. Surgery duration decreased significantly in CR and HPB procedures (*p* = 0.018 and *p* < 0.001). Estimated blood loss significantly decreased for CR operations (*p* = 0.001). The conversion rate decreased for HPB operations (*p* = 0.005). Length of hospitalization decreased for CR (*p* = 0.015) and HPB (*p* = 0.006) procedures. Oncologic quality, measured by histopathologic R0-resections, showed no significant changes. RoCS can be safely integrated into clinical practice without compromising patient safety or oncologic quality. It serves as an effective training pathway to guide robotic novices through their first steps in robotic surgery, offering promising potential for skill acquisition and career advancement.

## Introduction

Due to the exponential increase in robot-assisted procedures worldwide [1], including Germany [2], it has become necessary to train young, inexperienced surgeons in robotics during their residency [3]. Major oncologic and non-oncologic surgical procedures are increasingly being performed using minimally invasive techniques, and robotic-assisted procedures are among the state-of-the-art methods. Soon, robotics may significantly impact modern workplaces due to its technical advantages. Robotic-assisted procedures can offer numerous benefits, including the ability to achieve safe oncologic results.

However, robotic procedures remain a domain for experts with high surgical expertise. At present, training programs are limited to specific organ systems, and there is a need for more comprehensive training concepts [4–6]. Furthermore and when considering the rapid implementation of robotics in surgery and current teaching methods, residents are increasingly discontent [7]. Therefore, there is the urgent need for modern and more practical training for surgical residents.

In 2020, the Robotic Curriculum for young Surgeons (RoCS) was successfully implemented as a robotic training program for residents in the Department of General, Visceral, Vascular, and Transplant Surgery at the University of Magdeburg [8]. RoCS encompasses a comprehensive concept of perioperative process standardization, communication, organizational and personnel management, and surgical didactics [8]. The training program defines the surgeon’s intraoperative role as either bedside assistance or console assistance during visceral surgery [8]. Each procedural step is predetermined, with preoperative planning incorporated into the teaching concept for perioperative management [8]. RoCS enables each team member to contribute to the success of the robotic procedure and the team effort. However, the impact of RoCS on patient safety and outcomes remains unclear.

The objective of this study was to investigate the impact of such training program in terms of patient safety, perioperative and short-term patient outcome. It also aims to elucidate if the introduction of a training program leads to any disadvantages for the surgical and patient outcome.

## Material and methods

This study included all adult patients who underwent robotic procedures in the Department of General, Visceral, Vascular, and Transplant Surgery, Magdeburg, between the years 2017 and 2021, with a positive ethics committee vote. In order to assess the impact of RoCS, the study population was divided into two cohorts based on the date of RoCS implementation: Cohort 1 (before RoCS) consisted of patients who underwent robot-assisted surgery before RoCS was introduced in March 2020 and demonstrates the control group; while cohort 2 (after RoCS) included patients who underwent robotic surgery after RoCS was introduced and provided their consent. Cohort 1 (before RoCS) was analyzed retrospectively, cohort 2 (after RoCS) prospectively.

With the introduction of RoCS, novice surgeons have been provided with practical experience in both bedside and console assistant roles, thereby acquiring essential robotic surgery skills. The RoCS program is designed to ensure a stable learning environment, and one of its key components is the clustering of procedures according to the organ systems. Ongoing assessment of RoCS included evaluation of patient characteristics, intraoperative parameters, and outcome measures. Accordingly, the current study analyzes the impact of RoCS on patient outcomes based on the aforementioned organ-related clustering. A comparison of organ-related robotic procedures was conducted across the three main domains of visceral surgery: the upper gastrointestinal tract (UGI), colorectal (CR), and hepatopancreatobiliary (HPB) system. In order to eliminate heterogeneity and enhance comparability, any procedures that did not align with the organ-related clustering and could potentially cause significant bias within the organ-related subgroups were classified as “others” and excluded.

Data were collected using REDCap® (Vanderbilt University, 2004). The cohorts were analyzed descriptively based on patient demographics (sex, age, body mass index (BMI), American Society of Anaesthesiologists (ASA) classification, previous abdominal surgeries), surgical indication, and organ system. Perioperative parameters, including operative duration, estimated blood loss (eBL) and perioperative surgical complications of all organ-related groups were analyzed. In addition, postoperative parameters such as length of in-hospital stay (LOS), need for re-operation, in-hospital morbidity, and mortality were also analyzed. The follow-up analysis included parameters at 30 and 90 days after surgery and referred to unplanned re-admission, and consecutively 30d-/ 90d-morbidity, and/or mortality.

The statistical analysis was performed using SPSS® software package, Version 28 (IBM SPSS Statistics, Chicago, IL, USA). A significance level of 0.05 was established for all statistical tests. The pre-, intra-, and postoperative parameters, as well as the 30-day and 90-day outcome data for both cohorts, were compared and examined for significant differences. Categorical variables are presented as absolute (n) and relative (%) values. The comparison of both cohorts was conducted using Pearson’s χ2-Test or Fisher’s exact test if the count was lower than 5. Continuous variables are expressed as mean ± standard deviation (M ± SD). Continuous variables were compared using the Mann–Whitney U-Test.

## Results

A total of 367 robotic visceral procedures were performed between 2017 and 2021. As a result of the diversity and classification based on organ-related clustering, 16 of the 367 procedures were categorized as “others” and excluded from further analysis. This referred to one splenectomy, one partial peritonectomy, two TAPP, one ventral mesh rectopexy, two liver cyst enucleations, and five cholecystectomies performed in cohort 1. Four procedures were identified and categorized as “others” from cohort 2 (after RoCS) and were subsequently excluded: one exploration with peritoneal biopsy, one diaphragm and peritoneal metastasis resection, and two TAPP. Among the remaining 351 operations, 174 robotic procedures were identified between 2017 and March 19, 2020, and included in cohort 1 (before RoCS). Cohort 2 (after RoCS) included 177 patients who underwent robotic surgery between March 23, 2020, and December 29, 2021. A total of 192 UGI cases were identified, with 90 cases in cohort 1 and 102 cases in cohort 2. Two cases of hiatus hernia repair were excluded from follow-up due to unsuccessful contact attempts. In the HPB category, a total of 92 procedures were performed (before RoCS: *n* = 47); after RoCS: *n* = 45). For the CR subgroup, a total of 51 procedures were conducted, with 25 procedures before and 26 after the implementation of RoCS. In case of cohort 1 (before RoCS), only surgeons with an expert level of proficiency performed robot-assisted procedures using the robotic console. The robotic system for all procedures was the Si or Xi DaVinci© Surgical System.

### Cohorts, procedures and patient demographics

**Fig. 1:**
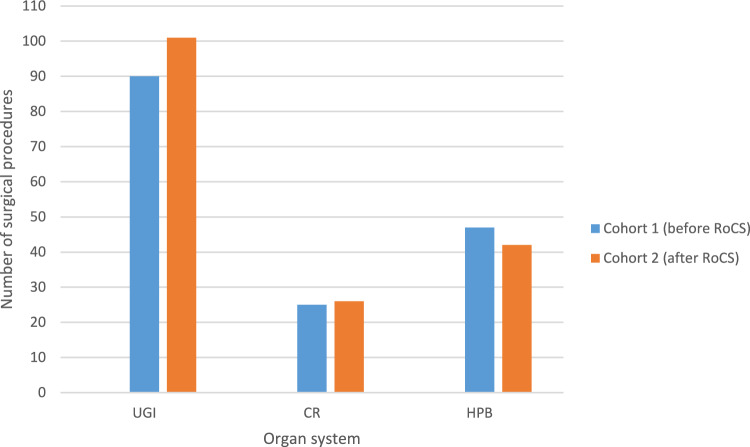
Overview of the caseload before (cohort 1) and after (cohort 2) implementation of RoCS, clustering referring to organ-related groups: upper gastrointestinal tract (UGI), colorectal (CR) and hepatopancreaticobiliary system (HPB)

The majority of robotic procedures were performed in the upper gastrointestinal tract (UGI), accounting for 54.7% (192/351) of both cohorts which is shown in Fig. 1.

As indicated in Table 1, there were no significant differences between cohort 1 and 2 (before and after RoCS) considering sex, age, BMI, ASA and rate of previous operations. Differences in the surgical indication were not significant (oncologic (56.3% and 62.1%) vs. functional (37.4% and 34.5%) vs. inflammatory (6.3% and 3.4%); *p* = 0.327). This also refers to the subgroups of UGI (*p* = 0.409), CR (*p* = 1.000) and HPB (*p* = 0.559) cases of both cohorts before and after RoCS.

**Table 1:** Overview of cohorts and patient demographics with comparison of cohort 1 vs. 2 (before vs. after RoCS implementation) and organ-related group (UGI, CR, HPB)

	Total			UGI			CR			HPB		
	Cohort 1 (before RoCS) (*n* = 174)	Cohort 2 (after RoCS) (*n* = 177)	*p-value*	Cohort 1 (before RoCS) (*n* = 90)	Cohort 2 (after RoCS) (*n* = 102)	*p-value*	Cohort 1 (before RoCS) (n = 25)	Cohort 2 (after RoCS) (n = 26)	*p-value*	Cohort 1 (before RoCS) (n = 47)	Cohort 2 (after RoCS) (n = 45)	*p-value*
	n (%) or M ± SD	n (%) or M ± SD		n (%) or M ± SD	n (%) or M ± SD		n (%) or M ± SD	n (%) or M ± SD		n (%) or M ± SD	n (%) or M ± SD	
Sex			0.749			0.385			0.572			0.529
Male	93 (53.4)	91 (51.4)	–	52 (57.8)	52 (51.0)	–	16 (64.0)	14 (53.8)	–	19 (40.4)	22 (48.9)	–
Female	81 (46.6)	86 (48.6)	–	38 (42.2)	50 (49.0)	–	9 (36.0)	12 (46.2)	–	28 (59.6)	23 (51.1)	–
Age (years)	61.3 ± 13.8	61.8 ± 14.0	0.761	61.5 ± 13.0	61.8 ± 13.1	0.835	60.0 ± 13.5	64.2 ± 14.7	0.300	63.1 ± 13.6	59.5 ± 15.7	0.248
BMI *(*kg/m^2^)^1^	28.0 ± 6.2	28.9 ± 7.0	0.229	29.3 ± 7.1	29.5 ± 7.7	0.953	26.4 ± 4.9	27.6 ± 5.9	0.496	26.4 ± 4.7	28.2 ± 6.3	0.204
ASA			0.278			0.585			0.621			0.847
1	11 (6.3)	7 (4.0)	–	2 (2.2)	1 (1.0)	–	3 (12.0)	1 (3.8)	–	4 (8.5)	5 (11.1)	–
2	101 (58.0)	98 (55.4)	–	55 (61.1)	59 (57.8)	–	16 (64.0)	17 (65.4)	–	22 (46.8)	21 (46.7)	–
3	61 (35.1)	67 (37.9)	–	32 (35.6)	38 (37.3)	–	6 (24.0)	8 (30.8)	–	21 (44.7)	18 (40.0)	–
4	1 (0.6)	5 (2.8)	–	1 (1.1)	4 (3.9)	–	–	–	–	–	1 (2.2)	–
Count of previous surgeries	0.6 ± 0.8	0.8 ± 0.9	0.080	0.7 ± 0.9	0.8 ± 1.0	0.249	0.6 ± 0.7	0.6 ± 0.8	0.907	0.7 ± 0.8	0.9 ± 0.9	0.263

The caseload of UGI procedures was stable when comparing cohort 1 (before RoCS) and 2 (after RoCS) (90/174, 51.7% vs. 102/177, 57.6%; *p* = 0.267) and when comparing the three subgroups of esophagus (E) (34/90, 37.8% vs. 47/102, 46.1%; *p* = 0.245), functional UGI (fUGI) (51/90, 56.7% vs. 47/102, 46.1%; *p* = 0.143) and stomach (5/90, 5.5% vs. 8/102, 7.8%; *p* = 0.529). Esophagectomies were performed as hybrid procedures with robotic abdominal and open thoracic approach.

Similar colorectal caseload was performed when comparing both cohorts (25/174, 14.4% vs. 26/177, 14.7%; *p* = 1.000). No significant differences were found between both cohorts regarding the type of colorectal procedure: right (6/25, 24.0% vs. 10/26, 38.5%; *p* = 0.368) and left hemicolectomy (2/25, 8.0% vs. 3/26, 11.5%; *p* = 1.000), sigmoid/anterior rectal resection (6/25, 24.0% vs. 4/26, 15.4%; *p* = 0.499) and low anterior rectal resection (10/25, 40.0% vs. 8/26, 30.8%; *p* = 0.565). In each cohort, one ileal/ colonic segmental resection was performed (1/25, 4.0% vs. 1/26, 3.8%; *p* = 1.000).

The caseload of HPB procedures showed no significant differences before and after RoCS (47/174, 27.0% vs. 45/177, 25, 4%; *p* = 0.808). Comparing liver (L) cases of cohort 1 vs. 2, the caseload differed without significance and included right (6/19, 31.6% vs. 8/34, 23.5%; *p* = 0.746) and left hemihepatectomies (4/19, 21.0% vs. 2/34, 5.9%; *p* = 0.172), and minor liver resections (9/19, 47.4% vs. 21/34, 61.8%; *p* = 0.391). Cohort 2 of the L procedures included three (8.8%) in-situ splits, cohort 1 none (*p* = 0.545).

In cohort 1 (before RoCS) significantly more pancreatic resections were performed in comparison with cohort 2 (28/174, 16.1% vs. 11/177, 6.2%; *p* = 0.004). The eleven procedures (100%) of cohort 2 (after RoCS) were distal pancreatectomies. Cohort 1 (before RoCS) included distal pancreatectomies in 89.3% (25/28) and three partial pancreatoduodenectomies (3/28; 10.7%).

### Intraoperative parameters

No significant differences were observed for UGI procedures regarding operation duration, as shown in Table 2. In both UGI groups, the majority of operations were performed without any intraoperative complications (80.0 and 82.4%, respectively). “Other” intraoperative complications of cohort 1 (before RoCS) referred to one thoracic duct leak, one conversion to laparoscopic approach due to technical issues, unsuccessful repositioning of the upside-down stomach with conversion to laparoscopic approach, altered diaphragm, macrohematuria, three pleural lesions and six incidents of bleeding. One pneumothorax, five pleural lesions, one emphysema due to trocar dislocation and five incidents of bleeding were observed in cohort 2. The overall conversion rate remained stable (4 vs. 4; *p* = 0.373).

**Table 2:** Intra- and postoperative parameters with comparison of cohort 1 vs. 2 (before vs. after RoCS implementation) referring to the organ-related group (UGI, CR, HPB)

	UGI			CR			HPB		
	Cohort 1 (before RoCS) (n = 90)	Cohort 2 (after RoCS) (n = 102)	*p- *value	Cohort 1 (before RoCS) (n = 25)	Cohort 2 (after RoCS) (n = 26)	*p- *value	Cohort 1 (before RoCS) (n = 47)	Cohort 2 (after RoCS) (n = 45)	*p- *value
	n (%) or M ± SD	n (%) or M ± SD		n (%) or M ± SD	n (%) or M ± SD		n (%) or M ± SD	n (%) or M ± SD	
Intraoperative									
Duration (min)	244 ± 126	273 ± 133	0.110	358 ± 99	294 ± 79	**0.018**	392 ± 117	297 ± 104	** < 0.001**
Estimated blood loss (ml)	228 ± 365	223 ± 260	0.054	361 ± 297	144 ± 125	**0.001**	608 ± 632	414 ± 395	0.278
Complications									
None	72 (80.0)	84 (82.4)	0.714	24 (96.0)	23 (88.4)	0.610	32 (68.1)	36 (80.0)	0.238
Organ lesion	5 (5.6)	6 (5.9)	1.000	–	–	–	1 (2.1)	–	1.000
Serosal lesion	1 (1.1)	4 (3.9)	0.373	–	1 (3.8%)	1.000	1 (2.1)	–	1.000
Re-resection/renewal anastomosis	2 (2.2)	2 (2.0)	1.000	–	–	–	3 (6.4)	2 (4.4)	1.000
others	14 (15.6)	12 (11.8)	0.444	1 (4.0)	2 (7.7)	1.000	10 (21.3)	7 (15.6)	0.594
Postoperative									
Length of stay (LOS) (days)	12.7 ± 13.6	14.0 ± 16.3	0.394	15.1 ± 12.1	8.8 ± 3.5	**0.015**	16.6 ± 12.2	12.0 ± 10.3	**0.006**
In-hospital morbidity (Clavien– Dindo ≥ 3)	19 (21.1)	18 (17.6)	0.585	4 (16.0)	1 (3.8)	0.191	9 (19.1)	8 (17.8)	1.000
In-hospital mortality	–	–	–	–	–	–	–	–	–
30 days- hospitalization / unplanned re-admission	2 (2.2)	4 (3.9)	0.686	2 (8.0)	3 (11.5)	1.000	4 (8.5)	1 (2.2)	0.362
30 days- morbidity (Clavien– Dindo ≥ 3)	1 (1.1)	2 (2.0)	1.000	2 (8.0)	–	0.235	4 (8.5)	1 (2.2)	0.362
30 days- mortality	1 (1.1)	–	0.469	–	–	–	–	–	–
90 days- hospitalization / unplanned re-admission	4 (4.4)	6 (5.9)	0.752	2 (8.0)	2 (7.7)	1.000	4 (8.5)	–	0.117
90 days- morbidity (Clavien– Dindo ≥ 3)	1 (1.1)	5 (4.9)	0.217	1 (4.0)	1 (3.8)	1.000	4 (8.5)	–	0.117
90 days- mortality	3 (3.3)	1 (1.0)	0.342	–	–	–	1 (2.1)	–	1.000

Bold values highlight relevant results

A significant reduction in surgical duration was observed in robotic CR procedures, as shown in Tab. 2. A high percentage of operations without any intraoperative complications was observed in both CR groups (96.0 and 88.4%, respectively). “Other” intraoperative complications during CR procedures included one bleeding incident (cohort 1) and one serosal tear und two bleeding incidents in cohort 2. No significant changes in the conversion rate was observed (2 vs. 1; *p* = 0.610).

The surgical duration of HPB procedures decreased significantly (*p* =  < 0.001). The percentage of HPB procedures without any intraoperative complications increased up to 80.0%, although this did not reach statistical significance (*p* = 0.238). “Other” intraoperative HPB complications of cohort 1 included ten events: seven instances of bleeding, two cases of pneumothorax and one patient experienced a repolarization abnormality in their ECG during surgery. In Cohort 2, seven other complications were documented: six bleeding events and one incident with injury of the diaphragm and pericardium. The conversion rate decreased significantly for HPB operations (16 vs. 4; *p* = 0.005).

### Postoperative parameters and patient outcome

There was a significant decrease in LOS for robotic CR and HPB procedures, as shown in Table 2. There were insignificant changes in the rehospitalization rate, morbidity, and mortality after 30 and 90 days for both cohorts. One UGI patient died within 30 days of hiatus hernia repair due to ischemic insult in combination with carotid dissection in cohort 1 (before RoCS). The cause of death in the UGI group after 90 days was related to the decompensation of pre-existing heart failure (*n* = 1), and multiorgan failure (*n* = 2). In cohort 2 of UGI (after RoCS), one patient died due to respiratory insufficiency following pneumocystic jirovecii pneumonia. One of the liver patients in cohort 1 (before RoCS) died within 90 days of the operation due to intracerebral hemorrhage.

### Histopathological quality

The histopathological R0-resection of malignancies showed a positive trend, although without statistical significance as shown in Table 3. The R0-resection rate for each organ-related subgroup increased up to 100% without significance. Table 3 also showed an absolute increase in liver metastasis resections from 37.5 up to 60%.

**Table 3:** Overview of histopathological malignant diagnoses and R0-resection rates with comparison of cohort 1 vs. 2 (before vs. after RoCS initiation) referring to organ-related groups of UGI, CR, and HPB

	Cohort 1 (before RoCS) n (%)	Cohort 2 (after RoCS) n (%)	*p* value
**UGI**	**34**	**47**	
Squamous cell carcinoma	13 (38.2)	18 (38.3)	
Adenocarcinoma	21 (61.8)	28 (59.6)	
Gastrointestinal stroma tumor	–	1 (2.1)	
R0-resection	32 (94.1)	46 (97.9)	0.566
**CR**	**20**	**20**	
Adenocarcinoma	18 (90.0)	19 (95.0)	
Neuroendocrine tumor	2 (10.0)	1 (5.0)	
R0-resection	20 (100.0)	20 (100.0)	1.000
**HPB**			
** Liver**	**16**	**25**	
HCC	7 (43.8)	6 (24.0)	
CCC	3 (18.7)	3 (12.0)	
Liver metastasis	6 (37.5)	15 (60.0)	
Intermediate-cell carcinoma	–	1 (4.0)	
R0-resection	14 (86.4)	25 (100.0)	0.146
** Pancreas**	**21**	**6**	
Adenocarcinoma	14 (66.7)	5 (83.3)	
Neuroendocrine tumor	6 (28.6)	1 (16.7)	
Solid pseudopapillary tumor	1 (4.7)	–	
R0-resection	19 (90.5)	6 (100.0)	1.000

Bold values highlight relevant results

## Discussion

Robotic procedures are becoming daily routine with an increasing caseload [1]. Therefore, safe implementation of a structured resident’s training is mandatory. The implementation of a robotic curriculum is both necessary and feasible in the early stages of training, without the requirement of specific fellowships [4, 9]. Currently, robotic training programs are limited to surgical specializations, such as UGI, HPB, CR, or single operations like cholecystectomy [10–13]. The primary objective of RoCS is to equip residents with basic robotic competence by engaging them in robotic procedures in the early stages of their surgical training. Therefore, RoCS is an educational intervention that is implemented regularly. According to the four-level model of training evaluation proposed by Kirkpatrick, it is crucial to implement the educational intervention within the workplace on a regular basis in order to perform the highest level of evaluation [14, 15]. This study aimed to investigate the effect of the implementation of the comprehensive training program RoCS on patient safety and surgical quality. Therefore, assessing the impact of RoCS on clinical patient outcomes represents the highest level of evaluating an educational intervention. However, it is not feasible to deduce recommendations for improving the training program specifically based on this evaluation [16].

### Patient outcome, safety and oncologic quality

This study did not show any elevated risk for the patients regardless of the organ system. Both cohorts had an equal number of cases of UGI and CR procedures, making UGI and CR ideal for assessing the impact of RoCS.

Rückbeil et al. implemented a modular in-house training concept for CR category with mentoring three trainees resulting in balanced patient outcome of 100 consecutive cases [17]. Thomas et al. implemented a comprehensive colorectal training program safely and effectively, yet only for two surgeons [18]. They could show comparable short-term survival and oncologic outcomes when comparing robotic to non-robotic approach [18]. Saqib et al. reviewed a single, newly appointed colorectal surgeons and significantly reduced operation time (2.5 h) and no intra- and postoperative complications as result of the learning curve [19]. They point out that a learning curve plateau can be achieved through combination of training and proctoring [19]. Aradaib et al. showed with the first 55 consecutive conducted colorectal resections performed by four surgeons with benign and malign indications that implementation of a robotic colorectal training program is safe [13]. Formisano et al. showed safe training of two senior attendings for 20 consecutive cases of right hemicolectomy [20]. Sian et al. showed that after European Academy of Robotic Colorectal Surgery (EARCS) training two surgeons had good oncologic quality (R0 margins) of 30 consecutive cases days for different colorectal resections [12]. Consistent with these previous studies, implementing our training program (RoCS) was associated with a significant decrease operation duration, eBL, LOS and non-significantly lower in-hospital morbidity. Nonetheless, it is crucial to recognize that these studies only encompassed a restricted number of surgeons and did not encompass the participation of residents. Sian et al. demonstrated no intraoperative complications [12], while RoCS showed a stable intraoperative workflow without complications in over 84%, even without statistical significance. In conclusion, no negative impact on patient safety regarding CR cases and involving residents in the early stages could be shown.

To the current status of knowledge, full robotic esophageal resection is a safe procedure with outcomes comparable to those of open or hybrid approaches [21, 22]. In this study, esophagectomies were part of the UGI group and performed as hybrid procedures. Accordingly, it is anticipated that substantial shifts in perioperative parameters will be evident following the implementation of routine full robotic resections. To fulfill the requirements of the Delphi Consensus for Robotic-assisted minimally invasive esophagectomy (RAMIE) training, comprehensive training programs must be provided at the home institution for residents [23]. RoCS is designed to address this gap not only in the context of esophagectomies, but also in bariatric surgery. Bellorin et al. highlighted in their multicenter study that short-term quality outcomes of bariatric surgery can be maintained even with a learning curve during a fellowship [24]. They concluded that adoption of robotics for sleeve gastrectomy is safe [24]. However, they restricted this conclusion to surgeons who have completed their residency. Straatman et al. demonstrated patient safety during proctored adoption for robotic hiatus hernia repairs of four surgeons [25]. In alignment with these studies, our patient outcome did not vary and patient safety was ensured while implementing training structures. Therefore, no enhanced risks for the patient could be shown due to consistent patient outcome and perioperative parameters.

Furthermore, intraoperative significant reduction of the operation time and the significant decrease in LOS of HPB procedures showed a positive effect for the patients while including residents. In addition, the increased oncologic quality in cohort 2 (100% R0 margins in liver resections) is superior to 5.3% positive margins in overall average described in the systematic review and meta-analysis of Rahimli et al. [26] McCarren et al. involved fellows after basic HPB training in their complex robotic procedures without challenging high surgical quality results [27]. One of the conclusion reached by the ROBOT4HPB conference highlighted the significance of employing a two-surgeon robotic method, comprising one surgeon at the console and at least one senior HPB resident at the bedside, in order to ensure patient safety [28]. In contrast to the aforementioned recommendation, in our institution all residents are engaged in robotics training, which allows them to develop basic proficiency in HPB procedures while under supervision. Farrugia et al. implemented a robotic HPB program safely with a step-up approach of surgical complexity of procedures [29]. From the very beginning of the program of Farrugia et al., three assistants and three theatre staff were members of the team and operating with two consultants [29]. This idea is reminiscent of our training approach, but it is exclusive to HPB.In conclusion, also for HPB procedures, our data demonstrated that patient safety and surgical quality is guaranteed when a training concept (RoCS) is introduced.

### Robotic training of young surgeons

It is suggested by the Orsi Consensus Meeting on European Robotic Training (OCERT) that international training pathways are required to successfully implement robotic surgery [30]. Acquiring basic robotic competence can be achieved through a combination of surgical exposure, didactic learning, and training [31]. To keep up with advancements of robotic surgical assistance systems and related increase in caseload, training during daily robotic procedures is essential for ensuring patient safety and surgical quality. RoCS is designed to meet specific needs within the German surgical training system while also having a transferable conceptual design [8]. RoCS involves residents from the onset of their surgical experience addressing the specific aspects of teaching basic procedural steps rather than complete procedures. Unlike specialized fellowships or training workshops, RoCS focuses on equipping novice surgeons with the ability to perform bedside assistance and subsequently progress to low-complex procedural steps while supervised. In essence, RoCS provides residents with the foundational skills required to become proficient in robotic surgery [8]. The process of acquiring basic robotic competence differs significantly from that of attaining expert status. Participation, in-house and simultaneous training with experts are the key to success [5, 17, 32]. Our research results demonstrates that the implementation of a structured training regimen does not detrimentally impact patients. Consequently, RoCS provides an secure environment for patients in which young surgeons can learn on a daily basis.

However, it should be noted that RoCS is a program confined to a single center. The current structural challenges resulting from hospital reform in Germany and modifications to training regulations have a significant impact on residency training and interfere with trainees’ needs. [33–35] To establish a uniform curriculum for Germany, it is necessary for the surgical society to take the lead, independently of any robotic system.

### Limitations

The limitations of this study relate to the retrospective nature of the control group (cohort 1, before RoCS). Inherently biased is the clustering of organ-related groups. Nevertheless, this approach provides a general overview of the distinct areas of robotic surgery. Furthermore, it is also conceivable that the observed improvement of clinical outcome before and after RoCS is partially attributable to enhanced clinical process management and standardization, rather than only to the aspect a comprehensive training concept. In addition, challenging circumstances, such as the COVID-19 pandemic, and the using surgical innovations daily [36] causes natural fluctuation of indications and variation of case loads over time. This is particularly relevant to this study, as the data analysis concludes in December 2021. Therefore, further follow-up analysis is mandatory. Moreover, the feasibility of the RoCS concept must be demonstrated through multicenter implementation and the integration of various robotic systems.

## Conclusion and perspective

The results of the present study indicate that the concept of RoCS and its implementation does not pose any risk to the patient, nor reduces oncologic quality and therefore, is safe. Furthermore, this study describes the positive impact of RoCS on intraoperative complications and patient outcomes. Nevertheless, improved patient outcome should be considered multifactorial. Incorporation of RoCS into routine surgical care requires standardization of perioperative settings, intraoperative workflow, surgical communication, and evaluation. Perioperative process management, including curricular teaching structures, provides opportunities for quality improvement. Despite a broad spectrum of robotic procedures, the implementation of a robotic training program during the early stages of surgical education is feasible.

In additionally, implementing a comprehensive training program, such as RoCS, addresses the needs and aspirations of young surgeons with respect to and despite their own learning curves. Structured routine training can have a major impact on young surgeons in terms of their career, gaining experience and developing their surgical technique. RoCS can provide a teaching environment while ensuring patient safety.

Due to the limited data regarding curricular training structures in robotic visceral surgery during work hours, further studies are needed to support trainees in becoming capable and qualified robotic surgeons. Real-world data from implemented training programs during daily routine will provide valuable insights. Follow-up data and multicentre analyses are also necessary to verify the impact and effects of robotic surgery training programs.

## Data Availability

No datasets were generated or analyzed during the current study.
